# Using Mendelian Randomization to Decipher Mechanisms of Bone Disease

**DOI:** 10.1007/s11914-018-0467-3

**Published:** 2018-09-10

**Authors:** Katerina Trajanoska, Fernando Rivadeneira

**Affiliations:** Departments of Internal Medicine and Epidemiology, Erasmus MC University Medical Center, Rotterdam, The Netherlands

**Keywords:** Osteoporosis, Bone mineral density, Fractures, Mendelian randomization, Review

## Abstract

**Purpose of Review:**

This review summarizes the basic principles of Mendelian randomization (MR) and provides evidence for the causal effect of multiple modifiable factors on bone outcomes.

**Recent Findings:**

Several studies using MR approach have provided support for the causal effect of obesity on bone mineral density (BMD). Strikingly, studies have failed to prove a causal association between elevated 25(OH) D concentrations and higher BMD in community-dwelling individuals.

**Summary:**

The MR approach has been successfully used to evaluate multiple factors related to bone mineral density variation and/or fracture risk. The MR approach avoids some of the classical observational study limitations and provides more robust causal evidence, ensuring bigger success of the clinical trials. The selection of interventions based on genetic evidence could have a substantial impact on clinical practice.

## Introduction

A fundamental goal of epidemiological (observational) studies is to determine causal factors of diseases. However, in observational studies, we test for association, which by itself does not imply causation. Two logical fallacies *cum hoc ergo propter hoc* (“with this, therefore because of this”) and *post hoc ergo propter hoc* (“after this, therefore because of this”) challenge the interpretation of observational studies. The relationship between exposure (expected cause) and disease (expected outcome) can be distorted by (a) the presence of unmeasured or unaccounted confounders, (b) reverse causation, and (c) a variety of other potential biases. Although proper study designs and analytical approaches can minimize the effect of the aforementioned factors, we still fail to account for most of them. Therefore, interventions based exclusively on evidence derived from association studies might turn out fruitless.

Some factors and biomarkers identified in observational studies have failed to be confirmed by large, robust randomized control trials (RCTs). For instance, in the past several years, observational studies [1–3] and one small RCT [4] have provided encouraging evidence for the beneficial effect of the vitamin D and calcium supplements on bone health. Thus, vitamin D and calcium supplementation have been included in the clinical guidelines for osteoporosis management and fracture prevention [5, 6]. However, in the past years, from a total of 38 RCTs (14 large and 24 small), the majority failed to detect a benefit of vitamin D and calcium supplements [7–9]. Moreover, a small proportion of the trials have found modest protective effects [10], and some have even shown an increased harm (e.g., falls, fractures) [11]. Even though experimental [randomized] studies are considered to be the gold standard for estimating causality in research [12], they have their own caveats like limitations due to ethical and technical issues and the exposure cannot be randomized, or being time-consuming and frequently costly [13]. Moreover, the lack of external validity (generalizability of the treatment/condition outcomes) affects the reliability of the results from the RCTs, which may result in flawed policy recommendations. In order to overcome the limitations from the observational and experimental studies, up till now, many methods (conditioning, mechanism-based, natural experiments) for causal inference have been developed that can be easily applied in epidemiological settings and can improve the identification of clinically relevant risk factors. Mendelian randomization is one of them. The aim of this review is to explain the basic principles of Mendelian randomization and provide examples of how Mendelian randomization has been applied to bone research.

## Casual Inference: Mendelian Randomization Analysis and Principles

**Fig. 1:**
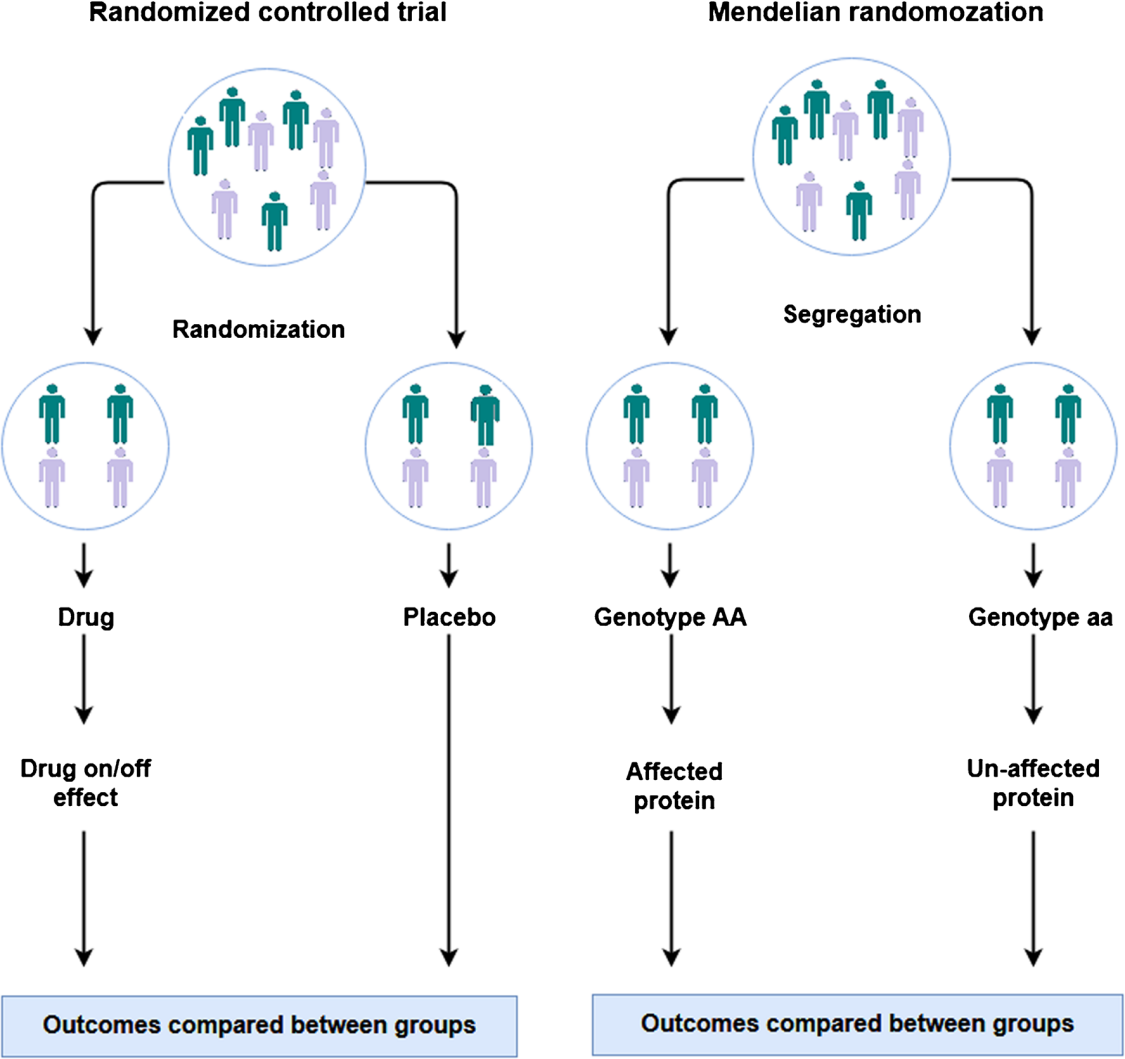
Comparison of the design of Mendelian randomization study and a randomized controlled trial

**Fig. 2:**
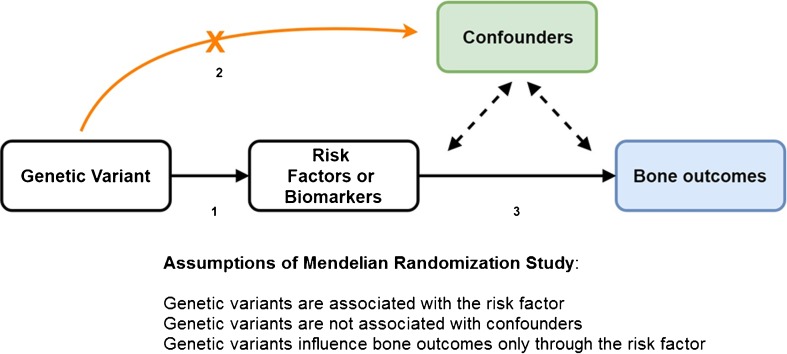
Directed acyclic graph (DAG) represents the relationship in a typical Mendelian randomization model

Mendelian randomization plays an important role in causal inference. During conception, parental gametes combine to form a zygote. Each gamete contains a different set of DNA as a result of recombination and independent assortment during conception resulting in genetically defined subgroups of individuals. The Mendelian randomization (MR) design is considered to be analogous to a RCT [14] where instead of random allocation of participants to interventions (treatments or preventive measures) individuals are randomized by nature according to carriership of gene variants that regulate susceptibility to a specific exposure (Fig. 1). Within both approaches, individuals are divided into random groups balanced across confounding factor(s). Following this principle, genetic variants associated with specific risk factors can be used as a non-confounded proxy to investigate the causal association between the risk factor in question and disease outcomes. Moreover, genetic variants have the advantages of being largely fixed since conception and remain stable throughout life. The expansion of genome wide association studies (GWAS) and improvement in array and imputations panels has enabled well-powered settings facilitating the identification of numerous genetic variants associated with different diseases and complex traits. Such large yield in genetic discoveries propelled by large-scale GWAS has improved considerably the extent of explained trait variance and the prospect of risk prediction of common diseases [15]. This also means that the MR approach leveraged by the abundance of genetic discoveries can now be easily implemented across numerous observational settings. This way, the MR approach can provide prior knowledge before launching RCTs or it can give way to more valid estimates of causal relationships in situations where an RCT cannot be conducted (e.g., smoking and alcohol intake). If MR findings provide evidence of causality for a specific marker, the next step will be to identify the correct biological pathway before performing RCTs. In addition, the MR is a simple and cost-effective method to assess causal relationships between risk factors and health outcomes. In order to obtain unbiased estimates, three key assumptions of MR need to be fulfilled with regard to the instrument: (1) genetic variants are associated with the risk factors or biomarkers under study; (2) genetic variants should not be associated with known confounding factors; and (3) it affects the outcome only through the risk factor and not via other biological pathways (pleiotropy) (Fig. 2). The first assumption can be easily verified by exploring the data. Even if this assumption holds, we need to test the strength of the association between the genetic variants and exposure (e.g., using partial *F* statistic) to avoid week instrument bias [16]. The second and third assumption can be difficult to address. First, we cannot estimate the association between the genetic variant and unobserved confounders (second assumption). However, we can still test the association with observed potential confounding factors or/and search the literature for any reported associations. Second, the presence of pleiotropy (third assumption) can be indirectly detected and corrected by using robust statistical methods [17] (e.g., MR-Egger regression, median weighted). In short, Egger regression assumes that the pleiotropic effect of the variant is independent of the phenotypic effect. If the pleiotropic effects act via a confounder of the “exposure-outcome” association, this assumption will be violated. Moreover, this will affect its associations with both the exposure and the outcome indicating the potential presence of directional pleiotropy. Finally, if the above assumptions hold, the MR can give reliable evidence for causation overcoming the typical pitfalls present in observational studies.

## Mendelian Randomization Debunks the Findings from Observational Studies: an Example

It is well established that heavy alcohol drinking during pregnancy has a serious effect on diverse health outcomes of the children [18]. Currently, there is no known safe level of alcohol that can be consumed at any time during pregnancy. However, many women do drink alcohol during pregnancy, generally at a moderate level, as a result of the conflicting messages from the health guidelines. Some of them promote complete abstinence while others recommend moderate drinking. These contradicting messages largely reflect the inconsistent findings from observational studies. For example, some observational studies have found that moderate drinking during pregnancy is even associated with a better cognitive function in children [19]. Nonetheless, the association can be confounded by many socio-economic factors. Taking all these confounders into consideration does attenuate the association, but does not eliminate the effect fully possibly due to residual confounding [20].

Alcohol is metabolized in the body by several alcohol dehydrogenase (ADH) enzymes. Variation in the genes that encode these enzymes influences the metabolic rate of alcohol [21]. Slow metabolizers will be exposed to higher alcohol levels for a longer time compared to fast metabolizers. Thus, it is hypothesized that alleles which increase the metabolism of ethanol will protect against abnormal brain development in infants [20] (as a result of less pronounced exposure to alcohol). For example, researchers of the Avon Longitudinal Study of Parents and Children (ALSPAC, UK-based children cohort) found that four genetic variants in alcohol metabolizing genes were related to low IQ at age 8 in children (carriers of the “slow” metabolizing alleles) whose mothers were drinking during pregnancy [22]. Moreover, in the same study, Zuccolo et al. [20] found the same association observed by previous studies when using the observational approach, i.e., moderate drinking is associated with increased IQ. However, using the MR method, they found that children of mothers genetically predisposed to drink less were better at school than children of mothers genetically predisposed to drink more [20]. This example illustrates the benefits of the MR approach; considering that most of the observational studies found associations in the same direction, the MR studies disproved them. MR becomes quite relevant in those scenarios where the association under study is confounded by multiple factors (alcohol and cognition in this case). Other examples include studies examining the causal role of CRP [23], lipoprotein (a) [24], and vitamin D levels [25] with different cardiovascular outcomes, or the association of homocysteine levels with diabetes mellitus [26].

## Mendelian Randomization in Bone Biology

The human skeleton is made of a dynamically growing tissue, essential for locomotion, structural support of soft tissues, and protection of organs. In addition, the skeleton exerts metabolic functions providing a mineral reservoir (primarily for calcium, but also for magnesium and phosphorus) and serves as a depository for cytokines and growth factors that upon release can exert local and systemic effects. Bones are constantly reshaped and renewed throughout the lifespan, through the processes of modeling and remodeling, which are under genetic and environmental control. Modeling occurs in growing bones from birth to the mid-20s, when peak bone mass is achieved. With aging, the imbalance in bone remodeling leads to loss of bone mass and deterioration of bone structure, which predispose to osteoporosis and fracture. An individual’s peak bone mass ultimately relates to lifetime risk of fracture (i.e., the higher the peak bone mass, the lower the risk). Yet, partitioning the genetic and environmental influences (risk factors) exerting an effect on bone throughout the lifetime is not trivial. The Mendelian randomization (MR) approach provides means to assess the influence of risk factors on osteoporosis outcomes, including fracture.

To date, the MR approach in the bone field has been applied predominantly to assess cause-effect relationships between different risk factors or biomarkers in relation to bone mineral density as outcome (Table 1). Among these body composition factors, inflammation markers and vitamin D levels are the most frequently investigated exposures. In particular, MR analyses have clearly reinforced the role of low BMI as an important risk factor for loss of bone mass [27••, 28, 29]. Similarly, late puberty [30] and type 2 diabetes and associated glycemic traits [31•] have been shown to exert modest causal effects on bone outcomes; in contrast, genetically increased inflammation markers [32, 33], phosphate [34] (very low powered), and higher urate levels [35, 36] had no causal effect on skeletal outcomes including fracture risk. A recent study has found a modest effect of heel BMD on type 2 diabetes and coronary heart disease, opening the door of evaluating deeper the endocrine function of the bone [37]. Notably, studies investigating the causal role of vitamin D and milk calcium intake showed no evidence of association [38, 39, 40•, 41].

**Table 1:** Systematic literature review of applications of Mendelian randomization using bone-related phenotypes

Exposure	Cohort(s)	Number	Genetic variant(s)	MR method	Unit	Casual effect estimate	*p*	Ref
Obesity	Avon Longitudinal Study of Parents and Children (ALSPAC)	7470 children	FTO marker: rs9939609 MC4R marker: rs17782313	Instrumental variable regression model, 2SLS	1 g change in BMC per 1 kg change in fat mass	TB-BMC, 0.02 (− 0.20, 0.15) UL-BMC, 0.46 (0.31, 0.61) LL-BMC, 0.55 (0.41, 0.68) LS-BMC, 0.48 (0.33, 0.63)	*p* = 0.0002 *p* = 0.03 *p* = 0.002 *p* = 2.3 × 10^−06^	[27••]
	Avon Longitudinal Study of Parents and Children (ALSPAC)	5221 children	77 SNPs associated with higher BMI	Instrumental variable regression model, 2SLS	SD change in BMD per SD increase in BMI	SK-BMD, 0.02 (− 0.20, 0.15) UL-BMD, 0.46 (0.31, 0.61) LL-BMD, 0.55 (0.41, 0.68) LS-BMD, 0.48 (0.33, 0.63) PE-BMD, 0.39 (0.34, 0.64)	*p* = 0.78 *p* < 0.001 *p* < 0.001 *p* < 0.001 *p* < 0.001	[28]
	Cross-sectional cohort of employees of the Electricity Generating Authority in Thailand	2154 adults	FTO marker: rs9939609	Instrumental variable regression model, 2SLS	1 g/cm2 change in BMD per 1 kg/m2 change in BMI	TH-BMD, 0.02 (0.00, 0.03) FN-BMD, 0.01 (0.00, 0.03) LS-BMD, 0.00 (− 0.01, 0.01)	*p* = 0.010 *p* = 0.014 *p* = NS	[29]
Inflammation	The Rotterdam Study	6386 adults	29 SNPs associated with CRP levels	Weighted genetic risk score	OR for fracture per 1 SD increase in CRP	Fracture, 1.00 (0.99, 1.00)	*p* = 0.23	[32]
	Summary data from two consortia (DIAGRAM consortium and GEFOS consortium)	hsCRP, N/A BMD, 32,961	20 SNPs associated with CRP levels	Published GWAS summary data, IVW and WM approach	1 g/cm2 change in BMD per 1 log mg/L change in total hsCRP	FA-BMD, − 0.02 (N/A) FN-BMD, − 0.04 (N/A) LS-BMD, − 0.04 (N/A)	*p* = 0.69 *p* = 0.22 *p* = 0.30	[33]
Type 2 diabetes	Summary data from two consortia (DIAGRAM consortium and GEFOS consortium)	T2D, 149,821 (34,840 cases, 114,981 controls) BMD, 32,961	37 SNPs associated with increased T2D risk	Published GWAS summary data, IVW approach	SD change in BMD per odds in T2D	FN-BMD, 0.03 (0.01–0.06) LS-BMD, 0.02 (− 0.01, − 0.05)	*p* = 0.017 *p* = 0.133	[31•]
Fasting glucose	Summary data from two consortia (MAGIC consortium and GEFOS consortium)	FG, 133,010 BMD, 32,961	33 SNPs associated with higher glucose levels	Published GWAS summary data, IVW approach	SD change in BMD per 1 mmol/L increase in GF	FN-BMD, 0.13 (0.02, 0.25) LS-BMD, 0.08 (− 0.04, 0.21)	*p* = 0.060 *p* = 0.064	[31•]
2-h glucose	Summary data from two consortia (MAGIC consortium and GEFOS consortium)	2hGlu, 133,010 BMD, 32,961	6 SNPs associated with glucose level	Published GWAS summary data, IVW approach	SD change in BMD per 1 mmol/L increase in 2hGlu	FN-BMD, 0.10 (0.02, 0.19) LS-BMD, 0.10 (0.01, 0.19)	*p* = 0.043 *p* = 0.046	[31•]
Vitamin D	Cross-sectional study of unrelated Chinese Han women	1824 women	10 SNPs associated with vitamin D levels	Instrumental variable regression model, 2SLS	1 g/cm2 change in BMD per 1 log ng/mL change in total 25OHD	TH-BMD, − 0.04 (− 0.13, 0.04) FN-BMD, − 0.04 (− 0.13, 0.03) LS-BMD, 0.05 (− 0.16, 0.06) P1NP, 0.09 (− 0.29, 0.09)	*p* = 0.326 *p* = 0.261 *p* = 0.384 *p* = 0.312	[39]
	Canadian Multicentre Osteoporosis study (CaMos)	2254 adults	rs2282679	Instrumental variable regression model, 2SLS	1 g/cm2 change in BMD per 1 SD change in DBP	FN-BMD, 0.002 (0.003, 0.007)	*p* = 0.43	[38]
	Summary data from three consortia (SUNLIGHT consortium, DIAGRAM consortium and GEFOS consortium)	Vitamin D, 42,274 BMD, 32,961 eBMD, 142,487	5 SNPs associated with vitamin D levels	Published GWAS summary data, IVW approach	1 SD change in BMD per 1 SD change in 25OHD (g/cm2 eBMD)	FN-BMD, 0.02 (− 0.03, 0.07) LS-BMD, 0.02 (− 0.04, 0.08) eBMD, − 0.03 (− 0.05, − 0.01)	*p* = 0.37 *p* = 0.49 *p* = 0.02	[40•]
Urate	Framingham Heart Study (FHS)	2501 adults	5 SNPs associated with urate levels	Instrumental variable regression model, 2SLS	1 g/cm2 change in BMD per 1 mmol/L change in urea levels	TF-BMD, − 0.29 (− 0.60, 0.01) FN-BMD, − 0.27 (− 0.58, 0.03) LS-BMD, 0.08 (− 0.32, 0.48)	*p* = 0.06 *p* = 0.08 *p* = 0.68	[35]
	Chinese Han individuals	1322 adults	18 SNPs associated with serum uric acid	Instrumental variable regression model, 2SLS	1 g/cm2 change in BMD per 1 mmol/L change in urea levels	TH-BMD, 0.19 (− 0.36, 0.74) FN-BMD, − 0.19 (− 0.42, 0.81) LS-BMD, 0.39 (− 0.26, 0.98) P1NP, 0.11 (− 1.54, 1.75) b-CTX, − 1.45 (− 3.44, 0.27)	*p* = 0.50 *p* = 0.53 *p* = 0.26 *p* = 0.10 *p* = 0.07	[36]
Phosphate	School-based cross-sectional study from Helsinki	183 children and adolescents	3 SNPs within the FGF23 gene	Instrumental variable regression model, 2SLS	1 g/cm2 change in BMD per 1 ng/L change in S-FGF23	TH-BMD, 0.6 (− 0.27, 1.53)	*p* = 0.17	[33]
Calcium milk intake	Summary data from two	BMD, 32,961	1 SNP associated with lactose intolerance	Published GWAS summary data, IVW approach	N/A	N/A	NS	[41]
Late puberty	Summary data from two consortia (ReproGen consortium and GEFOS consortium)	Puberty, 39,486 women, 55,871 men BMD, 32,961	331 SNPs associated with the onset of menarche 43 SNPs associated with age at voice break	Published GWAS summary data, IVW approach, MR-base	SD change in BMD per 1 year earlier onset of puberty	Age at menarche:		[32]
						FA-BMD, 0.09 FN-BMD, 0.12 LS-BMD, 0.17	*p* = 0.18 *p* = 0.06 *p* = 0.005	
						Age at voice break:		
						FA-BMD, 0.05 FN-BMD, 0.002 LS-BMD, 0.12	*p* = 0.70 *p* = 0.99 *p* = 0.0003	

*BMD*, bone mineral density; *BMC*, bone mineral content; *BMI*, body mass index; *FN*, femoral neck; *TH*, total hip; *LS*, lumbar spine; *FA*, forearm; *UL*, upper limbs; *LL*, lower limbs; *SK*, skull; *PE*, pelvis; *eBMD*, estimated BMD from ultrasound; *hsCRP*, highly sensitive C-reactive protein; *GWAS*, genome wide association study; *IVW*, inverse variant weighted; *WM*, weighted median; *SD*, standard deviation; *N/A*, not applicable; *NS*, not significant; *2SLS*, two-stage least square

## Vitamin D and Bone Mineral Density

Vitamin D is required for normal bone maturation, formation, and mineralization. Low levels of vitamin D result in hypocalcemia, hypophosphatemia, and hyperparathyroidism, which in turn can lead to impaired mineralization, bone loss, and low BMD levels. Severe lack of vitamin D is known to cause rickets (in children) and osteomalacia (in adults) [42]. Nevertheless, the influence of vitamin D on the etiology of low bone mass and the predisposition to develop osteoporosis is still unclear due to inconsistent results across clinical studies. These differences can be attributable to aspects of study design (e.g., study power, type of recruited population, or aspects affecting the vitamin D measurement, like season, thresholds, assays among others).

There are four SNPs found by GWAS to be strongly associated with 25(OH) D levels, mapping back to genes implicated in vitamin D synthesis, transport, or metabolism. These include rs2282679 in *GC* (association with 25(OH)D: *p* = 1.9 × 10^−109^), rs12785878 near *DHCR7* (*p* = 2.1 × 10^−27^), rs10741657 near *CYP2R1* (*p* = 3.3 × 10^−20^), and rs6013897 in *CYP24A1* (*p* = 6.0 × 10^−10^) [43]. The vitamin D-binding protein (DBP), a group-specific component of serum alpha globulin, is encoded by the GC gene and it serves as the principal protein carrier for vitamin D and its metabolites [44]. On the other hand, the *DHCR7* gene produces cholesterol, a substrate for vitamin D production. Finally, *CYP2R1* (encoding 25(OH) D synthesis) and *CYP24A1* (encoding 1α25(OH)2D inactivation) provide the active form of vitamin D.

Three studies have scrutinized if the relationship between vitamin D and BMD is causal. Leong et al. [38] have investigated the causal relationship between vitamin D-binding protein (DBP) levels and BMD using individual level data (*N* = 2254) from the Canadian Multicentre Osteoporosis Study (CaMos). In line with their observational results, they showed that DBP might not be a critical player in causal pathways potentially linking vitamin D to BMD. The authors also overcame the sample size limitations of the individual level setting by performing an additional analysis using summary data from the well-powered SUNLIGHT and GEFOS consortia where the null results remained consistent. Furthermore, Li et al. [39] using the four aforementioned vitamin D-associated SNPs found no evidence for a causal effect of vitamin D levels on BMD and bone turnover markers in a population of Chinese postmenopausal women (*N* = 1824). Finally, Larsson et al. [40•] using data from the GEFOS consortium and UK BioBank study have also recently found that vitamin D levels had no effect on DXA-measured BMD (*N* = 32,965). However, they observed that elevated vitamin D levels could exert a small decrease in estimated BMD derived from heel ultrasound (*N* = 142,487). Although the genetic variants have modest effects on vitamin D levels and explain small proportion of the trait variance, the aforementioned studies using summary level data were well powered to investigate causal associations. Very recently, we have also shown lack of a causal relationship between vitamain D levels and fractured risk, investigated in 37,857 cases and 227 116 controls [45].

These results should be interpreted with caution since the MR efforts have examined a linear relationship between vitamin D levels and BMD. Possible threshold-dependent effects (effects present only in a subgroup with low vitamin D levels) are not examined by this approach. Extreme deficits in vitamin D are known to influence bone metabolism and result in disease (i.e., rickets, osteomalacia). In contrast, the MR setting is drawn in the general population, typically including relatively healthy elderly adults, so the findings might not be applicable to very old and frail people where vitamin D deficiency is frequently present. Another aspect relates to gene x environment (GxE) interactions, which can be challenging to consider in casual inference analyses. It has been postulated that vitamin D levels may be subject to GxE interactions [46, 47]. However, these interactions remain difficult to detect (as testing requires very large sample sizes which are not yet available). Until then, detecting the main effect of a genotype will be more reliable than testing for GxE interactions [48]. Either way, once GxE interactions are detected demonstrating that the exposure differs quantitatively between individuals, then the MR should be restricted to the specific subgroups where the environmental exposure is homogeneous.

## Limitations

In order to obtain unbiased estimates of causality, all three crucial assumptions of MR must be fulfilled. However, the verification of the assumptions is difficult, particularly assessing canalization and pleiotropic effects. In general, the results of MR are said to be robust when multiple methods to assess the MR assumptions are applied and the observed effects still stand. Most importantly, the interpretation of MR studies should be made with caution and with substantial knowledge of the underlying biology. There are multiple factors that can bias the estimates of MR studies: (1) *Insufficient power*—i.e., the probability that the null hypothesis can be rejected in the presence of true association between the biomarker and disease. If the genetic instrument explains a small proportion of the trait variance, a sufficiently powered sample size will be required to detect effect and sample size calculations should be performed and considered for the interpretation of the findings. (2) *Weak instrument bias*—strong instruments will force the association to be independent of confounders. With weak instruments, confounders are not equally balanced between genotype groups and the confounders can explain a given difference in phenotype more strongly than the instruments. Therefore, the instrument should be robustly associated with the exposure of interest. Similarly, an instrument may lack sufficient power when the outcome is only affected by large changes in the exposure. This is particularly relevant for complex traits where common genetic variants typically have a small effect. Therefore, the combined use of multiple variants as instruments will be warranted to achieve sufficient power. (3) *Pleiotropy—*i.e., when a gene or variant is associated with multiple traits. Even in the presence of a causal effect, the effect can still be due to other factors controlled by the genetic effect. Pleiotropy can be vertical (when genetic variants influence other factors downstream of the primary trait) and horizontal (when the genetic variants influence multiple traits separately). This is nicely illustrated in recent work examining the influence of adiposity and BMD [27••], where an effect on BMD can be mediated by fat mass, lean mass, or both, drawing the need for careful interpretation of the findings. (4) *Population stratification*—i.e., differences in genetic structure between subpopulations masked in the population under investigation. The genetic association between the instrument and the outcome should not be driven (or attenuated) by population stratification. Other potential ethnic differences between the discovery (exposure) and the target (outcome) settings (i.e., allele frequencies, linkage disequilibrium structure) should be considered in the interpretation of the MR findings as they reduce the strength of genetic instruments. (5) *Canalization/developmental compensation* due to operation of compensatory processes during development that may resist the phenotypic changes that result from the genetic variants being used as an instrument.

## Clinical Implication

The major advantage of the MR approach is that it can help overcome the expensive costs of running an RCT, by helping in the prioritization of interventions directed towards causal pathways. The selection of interventions based on genetic evidence could have a substantial impact on clinical practice with major considerable utility in primary prevention. In cardiovascular epidemiology, for example, PCSK9 (protein which influences LDL-C levels) has been identified as a potential drug target using MR methods [49]. Recently, phase II clinical trials have proven the safety and efficacy of the monoclonal PCSK9 antibodies [50]. Furthermore, the strengthening of the causal relationship between modifiable exposures and a wide range of outcomes related to complex diseases can help us improve the drug target identification and validation processes, i.e., the MR approach will contribute to robust determination of the role of factors within biological pathways. For example, a recent study has illustrated how drug mechanisms with genetic support are shown to succeed twice as often as those without it (from phase I to approval) [51]. In fact, this is the case for osteoporosis drugs as the highest degree of genetic support for drug target indications was related to the musculoskeletal (BMD), metabolic, and blood categories [52]. In addition, MR can help in identifying adverse effects and drug repurposing [53]. For example, it has been widely recognized that statins, commonly used for prevention of CHD, increase the risk of type 2 diabetes [54]. After the clinical trials, using MR approach, it has been shown that the risk of type 2 diabetes can be partially explained by inhibition of the HMGCR gene (produces enzyme targeted by statins) [55]. In principle, this example illustrates (in retrospective) the potential of the MR approach to inform RCT before their execution.

Looking back to bone-related phenotypes, most MR methods have evaluated the causality of specific exposures. To date, there are no studies that have investigated the causality of specific drug targets for osteoporosis. One novel osteoporotic treatment is the use of Romosozumab, a monoclonal antibody that targets sclerostin. However, recent trials have shown that Romosozumab is associated with (small yet real) increased risk of cardiovascular adverse events. This way, MR studies are warranted to evaluate the causal relation of Romosozumab treatment with this adverse effect, by investigating whether variations in the *SOST* gene are associated with cardiovascular or other adverse events.

## Conclusions

The Mendelian randomization (MR) approach is a robust strategy to determine causal relationships between risk factors and diverse health-related outcomes, including bone health. While still in its infancy, the MR approach has been used to evaluate multiple factors mostly related to bone mineral density variation and a few for fracture risk. Given the advent of large-scale GWAS identifying hundreds to thousands of genetic variants robustly associated with bone traits, together with the clear benefits of the MR approach to prioritize interventions of RCT, repurpose existing medications, and prediction of adverse effects, it is expected that many of the unsolved epidemiological questions of observational studies will be solved and better treatments for patients will emerge in the clinic.
